# Advances in Porous Structure Design for Enhanced Piezoelectric and Triboelectric Nanogenerators: A Comprehensive Review

**DOI:** 10.1002/gch2.202400224

**Published:** 2024-11-25

**Authors:** Zhassulan Turar, Merey Sembay, Assem Mubarak, Ayaulym Belgibayeva, Long Kong, Gulnur Kalimuldina

**Affiliations:** ^1^ Department of Mechanical and Aerospace Engineering Nazarbayev University Kabanbay Batyr Ave. 53 Astana 010000 Kazakhstan; ^2^ National Laboratory Astana Nazarbayev University Kabanbay Batyr Ave. 53 Astana 010000 Kazakhstan; ^3^ Xi'an Institute of Flexible Electronics (IFE) Northwestern Polytechnical University Xi'an Shaanxi 710129 China

**Keywords:** energy harvesting, piezoelectric nanogenerator (PENG), porous materials, self‐powered sensors, triboelectric nanogenerator (TENG)

## Abstract

Porous structures offer several key advantages in energy harvesting, making them highly effective for enhancing the performance of piezoelectric and triboelectric nanogenerators (PENG and TENG). Their high surface area‐to‐volume ratio improves charge accumulation and electrostatic induction, which are critical for efficient energy conversion. Additionally, their lightweight and flexible nature allows for easy integration into wearable and flexible electronics. These combined properties make porous materials a powerful solution for addressing the efficiency limitations that have traditionally restricted nanogenerators. Recognizing these benefits, this review focuses on the essential role that porous materials play in advancing PENG and TENG technologies. It examines a wide range of porous materials, including aerogels, nano‐porous films, sponges, and 2D materials, explaining how their unique structures contribute to higher energy harvesting efficiency. The review also explores recent breakthroughs in the development of these materials, demonstrating how they overcome performance challenges and open up new possibilities for practical applications. These advancements position porous nanogenerators as strong candidates for use in wearable electronics, smart textiles, and Internet of Things (IoT) devices. By exploring these innovations, the review underscores the importance of porous structures in driving the future of energy harvesting technologies.

## Graphical abstract

This article explores the latest advancements in piezoelectric and triboelectric nanogenerators (PENG and TENG) with a focus on porous materials. Key fabrication techniques, material properties, and performance improvements are discussed, highlighting the role of porosity in enhancing charge generation and energy conversion efficiency. Practical applications in wearable electronics and sustainable energy solutions are also presented.

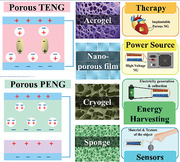

## 1 Introduction

Facing a critical need for new technologies due to an escalating energy crisis and worsening environmental impact, researchers are seeking alternative energy solutions to cut reliance on fossil fuels.^[^ ^1^, ^2^ ^]^ Energy harvesting has emerged as a core focus, intended to collect ambient energy from a range of sources and transform it into electrical power.^[^ ^3^, ^4^, ^5^ ^]^ Among energy harvesting techniques, piezoelectric nanogenerators (PENG) and triboelectric nanogenerators (TENG) show strong potential for converting mechanical energy into electricity.^[^ ^6^, ^7^, ^8^ ^]^

Despite their potential, PENG and TENG face limitations related to their structural and material components, which directly affect their electrical performance.^[^ ^9^, ^10^ ^]^ Particularly, PENG and TENG typically produce electrical power at milliwatt or microwatt levels, which may be insufficient for certain applications requiring higher power output.^[^ ^11^, ^12^, ^13^ ^]^ Therefore, improving power generation efficiency while maintaining device scalability remains challenging.^[^ ^14^ ^]^ Researchers have reported various approaches to overcome these limitations, including the integration of nanomaterials like carbon nanotubes and graphene oxide, as well as the exploration of organic polymers for enhanced flexibility.^[^ ^15^, ^16^, ^17^ ^]^ Furthermore, innovative techniques such as combining nanotechnology and printing technology have enabled the production of flexible electronics with improved surface area and mechanical properties.^[^ ^18^, ^19^ ^]^

Another emerging approach to address the challenges facing PENG and TENG lies in the utilization of porous structures.^[^ ^20^, ^21^, ^22^ ^]^ Porous materials are solids with voids filled with air or other gasses inside them; these include closed‐ and open‐cell foams and fiber‐woven and non‐woven materials.^[^ ^14^ ^]^ They offer unique advantages, including high surface area‐to‐volume ratios, which enhance electrostatic induction and promote greater charge accumulation.^[^ ^23^, ^24^, ^25^, ^26^ ^]^ Furthermore, the porous structure of porous framework material allows for the introduction of various chemicals throughout the entire volume of the material, enabling the creation of composite materials with unique properties.^[^ ^27^ ^]^ Recent advancements in porous TENG and PENG materials have shown promise in improving energy conversion efficiency, thereby bolstering their viability for practical applications.^[^ ^28^, ^29^, ^30^, ^31^ ^]^ Ongoing efforts to integrate these technologies into textiles show promise in empowering wearable electronic devices for various applications, including smart clothing, fitness trackers, and the Internet of Things (IoT) applications.^[^ ^32^, ^33^, ^34^ ^]^

Existing review papers in the field have provided valuable insights into the advancements and challenges in nanogenerator technology.^[^ ^35^, ^36^ ^]^ These reviews have covered topics ranging from the fundamental principles of energy harvesting to the latest developments in materials science and fabrication techniques.^[^ ^37^, ^38^, ^39^ ^]^ However, while previous reviews have laid a foundation for understanding the field, there remains a need for a comprehensive analysis of recent research focusing specifically on the role of porous structures in enhancing the performance of PENG and TENG.

This review provides a comprehensive overview of innovative advancements in nanogenerator technology, focusing on porous structures. By analyzing existing literature and highlighting recent research trends, the review elucidates the potential of porous structures to enhance the performance and efficiency of PENG and TENG. It examines electrostatic phenomena in detail and explores the specific impact of porous surfaces on charge generation to strengthen the technical understanding of these advancements. Additionally, the review underscores the importance of porous nanogenerators in meeting growing energy demands and advancing sustainable energy production, illustrating their advantages and applications in diverse fields, including wearable electronics, environmental sensing, and medical devices, with specific examples and potential use cases.

## 2 Working Principle of Porous PENG and TENG

Pore size and shape, which are directly affected by the synthesis process and structural design, have led to different classifications depending on the measurement range.^[^ ^40^ ^]^ For instance, Duat et al. categorize pores as micro‐pores when they are smaller than two nm, meso‐pores when they are below 50 nm and macro‐pores when they exceed 50 nm.^[^ ^41^ ^]^ In contrast, Rastegardoost et al. define classifications as follows: pico‐pores for sizes under one nm, nano‐pores from one nm to one µm, micro‐pores from one µm to 65 µm, meso‐pores between 65.5 µm and four mm, and macro‐pores ranging from 4 to 256 mm.^[^ ^42^ ^]^ The presence of pores affects materials in several ways, such as increasing electrical resistance and enhancing deformability.^[^ ^41^ ^]^ The next subsections provide an examination of the working principles of PENG and TENG, with a focus on how porosity affects their performance and efficiency.

### 2.1 Working Principle of Porous PENG

**Figure 1:**
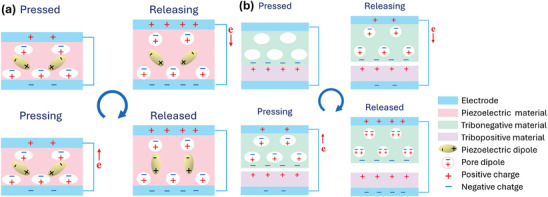
Working mechanism of porous a) PENG and b) TENG.

PENG operates on the principle of the piezoelectric effect, where electricity is generated in response to mechanical stress applied to a piezoelectric material. When external strain is applied to two electrodes with balanced Fermi levels on the material, a piezo potential difference is created between the internal and external Fermi levels at the contacts, causing charge carriers to flow through the external load to balance this disparity (**Figure** **1**a).^[^ ^12^, ^19^, ^20^ ^]^ Alternatively, applying an electric field to the piezoelectric material can induce mechanical strain.^[^ ^12^ ^]^

Porous structures significantly enhance the performance of PENG by offering several advantages over bulk materials. First, the increased surface area in porous materials allows for greater interaction between the material and applied strain, leading to higher energy output.^[^ ^42^ ^]^ The structural stability and mechanical flexibility of porous materials also result in a larger piezoelectric response under applied stress.^[^ ^43^ ^]^ Furthermore, the lower modulus and reduced volume of porous materials enable greater compression strains and significantly enhance piezoelectric output, all while minimizing size‐related limitations.^[^ ^43^ ^]^ This makes porous‐structured PENG highly effective for various applications, where both flexibility and energy efficiency are critical.^[^ ^44^ ^]^

### 2.2 Working Principle of Porous TENG

TENG function by combining contact electrification and electrostatic induction, where charge transfer occurs at the interface of two contacting materials, and subsequent motion induces a potential difference (Figure 1b).^[^ ^24^, ^25^ ^]^ The primary operational modes of TENG include vertical contact‐separation, where surfaces move perpendicularly to each other; lateral sliding, involving parallel movement of surfaces; single‐electrode mode, which utilizes one grounded electrode; and freestanding triboelectric‐layer mode, in which a triboelectric layer operates independently to generate charge without direct attachment to electrodes.^[^ ^27^, ^28^, ^29^ ^]^

Porous materials play a crucial role in enhancing TENG performance by increasing the contact area, introducing internal air gaps, and improving charge trapping efficiency. These factors significantly boost output and make TENG more versatile in various applications, especially in environments requiring additional thermal, acoustic, or electromagnetic resistance.^[^ ^42^ ^]^ Furthermore, the effect of void cells in porous structures during the pressing and releasing cycle modulates the compression and enhances triboelectric performance by altering the contact area and charge distribution.

## 3 Porous Structures for Enhancing PENG and TENG Performance

**Figure 2:**
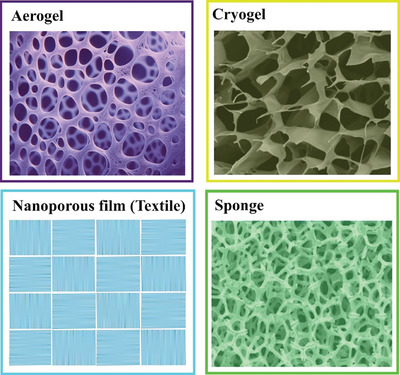
Different porous designs for the PENG and TENG: Material designs including aerogels, cryogels, nano‐porous film, and sponge.

Various effective strategies have been implemented to enhance the performance of PENG and TENG, addressing diverse energy harvesting and tactile sensing requirements across multiple applications. These strategies include selecting suitable materials, optimizing structural designs, and refining fabrication processes.^[^ ^45^ ^]^ One prevalent technique to enhance the output of PENG and TENG involves introducing micro‐ and nano‐scale pores in the materials, creating aerogels, cryogels, nano‐porous films, and sponges with a porosity over 90% (**Figure** **2**).^[^ ^30^, ^32^, ^33^, ^35^ ^]^ Different drying techniques produced materials with unique structures: aerogels had a fine, branched network with pores smaller than 200 nm,^[^ ^30^ ^]^ while cryogels had larger pores, a few microns in size, with smooth and continuous pore walls.^[^ ^33^ ^]^

These materials, characterized by their unique structural attributes, offer substantial improvements in piezoelectric and triboelectric performances, making them highly suitable for various practical applications, including wearable electronics and environmental sustainability.^[^ ^46^ ^]^ Thus, the porous structures for PENG reduce the breakdown voltage, hinder pre‐poling of the piezoelectric layer, and increase flexibility, thereby leading to larger strains and higher voltage outputs under similar forces.^[^ ^47^, ^48^ ^]^ On the other hand, the porous structures for TENG minimize the effective thickness, improve the surface roughness, and enhance electrostatic responses within pores, resulting in robust triboelectric outputs. **Table** **1** summarizes the key features and performance outcomes of different porous structure nanogenerators.

**Table 1:** Fabrication Techniques and Performance Characteristics of Diverse Porous Material Designs in PENG and TENG Applications.

Material	Synthesis method	Structural features	Type of nanogenerator	Performance				Application	References
				External force	V_OS_	I_SC_	Power density		
Aerogels									
TOCN	cross‐linking, curing, freeze‐drying	nanofibrils composed of dozens of cellulose molecular chains	PENG‐TENG	40 N, 1 Hz	104 V	8.3 µA	156 mW m^−2^	human motion energy: part of clothing, shoes self‐powered smart door panels liquid trace analyzer sound electric‐conversion for powering mini electronics sound energy harvesting	[51]
TOCN/MoS_2_		aerogel with nanofibrils and nanosheets	PENG	50 N, 1 Hz	43 V	1.1 µA	129 mW m^−2^		[52]
TOCN/PDMS		entangled, highly porous, and nanofiber‐like	PENG	0.05 MPa, 10 Hz	60.2 V	10.1 µA	6.3 mW m^−2^		[50]
TOCN/CNT		aerogel channels, forming a stable porous network	TENG	9 Ω	160 V	10 µA	2200 mW m^−2^		[45]
BC/HEC		large stacked layers with small pores	TENG	10 N	21 V	0.39 µA	7.91 mW m^−2^		[48]
MWCNTs/PVDF‐TrFE		interconnected tiny voids in the porous aerogel	PENG‐TENG	115 dB, 150 Hz	34.4 V	1.74 µA	11.62 mW m^−2^		[30]
PVDF‐TrFE		porous aerogel film	TENG	0.75Hz	105.6 V	–	9.9 mW m^−2^	energy harvesting from human motion	[54]
Cryogels									
C03t4‐Ag/Au	UV‐radiation and in situ reduction	larger pores sizes, rapid fabrication process	TENG	50 N, 5 Hz	233.85 V	23.6 µA	24.33 mW m^−2^	energy harvesting	[55]
Cr‐3t18	facile freezing, ultraviolet radiation, and thawing processes	hydrophobic moiety and nonsticky cryogels	TENG	50 N, 5 Hz	160 V	17 µA	2.95 mW m^−2^	energy harvesting	[56]
CNF‐based cryogel/MIL‐125	cross‐linking, freeze‐drying	the filtration efficiency and sensing/catalytic degradation	TENG	–	70.5 V	11.3 µA	–	intelligent wearable devices, smart medical applications	[57]
Nano‐porous films									
PVDF/BTO	electrospinning, nano‐catalyst‐assisted ink spray coating, soft‐template‐assisted synthesis	formation of nano‐porous structure, highly strained lattice structure	PENG‐TENG	15 N 5 N	444 V 124 V	19.02 mA m^−2^ 11.52 µA	105.6 mW m^−2^ 400 mW m^−2^	harvesting biomechanical energy, advanced electronic devices and sensors, intelligent bioelectronics	[33, 34]
BCZT films			PENG	2.7N	57.5 V	0.1 µA	–		[58]
NPCO/Mxene			TENG	10 N, 5 Hz	1680 V	–	10 400 mW m^−2^		[59]
Sponge									
PVDF/ZnO	casting and etching	Meso‐porous sponge‐like structure	PENG	1.58 N, 4 Hz	69.4 V		407 mW m^−2^	wearable piezoelectric nanogenerators for electronic devices	[35]
PVDF‐TrFE/PEO	electrospinning	thick sponge‐like fiber mats	PENG	1.58 N, 4 Hz	69.4 V	1.17 µA	407 mW m^−2^		[36]
PANI	dilute solution polymerization process	conductive elastic sponge	TENG	50% deformation, 6 Hz	520 V	6.3 µA	175 mW m^−2^	detection of NH_3_ leakage	[60]

Abbreviations: TOCN: 2,2,6,6‐Tetramethylpiperidine 1‐oxyl (TEMPO) oxidized cellulose nanofibril; PDMS: Polydimethylsiloxane; BC: Bacterial cellulose; HEC: Hydroxyethyl cellulose; PVDF‐TrFE: Poly(vinylidene fluoride‐trifluoroethylene); MWCNTs: Multi‐walled carbon nanotubes; BTO: Barium titanate (BaTiO₃); BCZT: Barium calcium zirconium titanate ((Ba₀.₈₅Ca₀.₁₅)(Ti₀.₉Zr₀.₁)O₃); NPCO: Nano‐porous cobalt oxide; PEO: Polyethylene oxide; PVDF: Polyvinylidene difluoride; CO3t4: Cryogel based on lauryl acrylate‐acrylamide (LA‐AAm); Cr‐3t18: Cryogel with 3% cross‐linker at 18 °C; CNT: Carbon nanotubes; PANI: Polyaniline nanowires.

### 3.1 Aerogel Films

Aerogels have become one of essential porous materials in the field of nanogenerators. They possess remarkable characteristics such as ultra‐lightweight nature, high specific surface area, and extremely low thermal conductivity, making them excellent candidates for piezoelectric and triboelectric applications in contrast to the traditional dense films.^[^ ^37^ ^]^

A typical procedure for the fabrication of aerogel films includes preparation of suspensions based on gel‐making nanofibrils, gel formation induced by a cross‐linker (glutaraldehyde), curing, freeze‐drying under vacuum or controlled atmosphere, and compression under a certain pressure (0.5 MPa).^[^ ^49^, ^50^ ^]^ Prepared aerogel films possess an interconnected, highly porous, and nanofiber‐like structure with nano‐sized pores.

**Figure 3:**
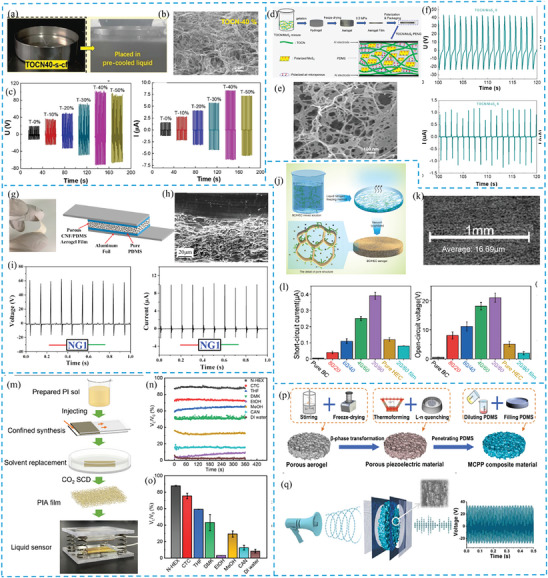
a) Freezing process of TOCN‐40% dispersion. b) SEM of TOCN‐40%. c) Open‐circuit voltage and short‐circuit current. d) Preparation route scheme. e) SEM of TOCN/MoS_2_ 6 aerogel pre‐compression. f) Open‐circuit voltage and short‐circuit current. g) Photograph and schematic of flexible porous CNF/PDMS aerogel film‐based NG device. h) SEM of porous CNF/PDMS NG film. i) Output voltage and current signals. j) BC/HEC aerogel preparation. k) SEM of BC/HEC 20/80 aerogel's porous structure. l) Voltage and current output. m) PIA film preparation and liquid analyzer design. n) Voltage–time curve for liquid droplets. o) Voltage error at first peak (0.5 s). p) MCPP composite preparation. q) Schematic of aerogel vibration testing by LDV and optimal voltage output. (a–c) Reproduced with permission from,^[^ ^51^ ^]^ Copyright 2024, Elsevier. (d–f) Reproduced with permission from,^[^ ^52^ ^]^ Copyright 2024, Elsevier. (g–i) Reproduced with permission from,^[^ ^50^ ^]^ Copyright 2024, Elsevier. (j–l) Reproduced with permission from,^[^ ^48^ ^]^ Copyright 2024, American Chemical Society. (m–o) Reproduced with permission from,^[^ ^47^ ^]^ Copyright 2024, American Chemical Society. (p,q) Reproduced with permission from,^[^ ^30^ ^]^ Copyright 2024, Elsevier.

One of the most promising examples of aerogel is 2,2,6,6‐tetramethyl‐1‐piperidinyloxy (TEMPO)‐oxidized cellulose nanofibrils (TOCN) (**Figure** **3**a,b), with their large aspect ratio and length, offering a substantial specific surface area (SSA) of 134.1 m^2^ g^−1^.^[^ ^51^ ^]^ When measured under open circuit conditions, the voltage and current reach 104 V and 8.3 µA respectively (Figure 3c), while the power density reaches up to 156 mW m^−2^.^[^ ^51^ ^]^ The inherent high SSA and porosity of TOCN aerogels enhance electrostatic responses within pores, resulting in robust triboelectric outputs. Despite the possibility of being used directly, TOCN aerogels are often combined or coated with different materials to solve structural fragility issues and further improve their piezoelectric and triboelectric properties. For example, when combined with MoS_2_, TOCN‐based aerogel piezoelectric films (Figure 3d,e) exhibits an open‐circuit voltage of 43 V, a short‐circuit current of 1.1 µA (Figure 3f), and a surface power density of 1.29 µW cm^−2^.^[^ ^52^ ^]^ This performance is comparable to conventional high‐performance piezoelectric polymers and surpasses that of the majority of cellulose‐based PENG. On the other hand, when coated with a polydimethylsiloxane (PDMS) by vacuum filtration, more remarkable piezoelectric outputs can be obtained (Figure 3g,h), such as an open‐circuit voltage of 60.2 V, a short‐circuit current of 10.1 µA, and a corresponding power density of 6.3 mW cm^−3^.^[^ ^50^ ^]^ A similar trend is observed for triboelectric TOCN aerogels, where incorporating a heterointerface with carbon nanotubes (CNTs) enhances structural stability and achieves a 40‐fold increase in compressive performance, maintaining a high porosity of 97.23%.^[^ ^45^ ^]^

Another way of improving cellulose aerogel is hybridizing it by adding substances that introduce new functional groups to the material. Luo et al. incorporated bacterial cellulose (BC) and hydroxyethyl cellulose (HEC) to create a hybrid aerogel (Figure 3j,k) that stands out for its biocompatibility and enhanced triboelectric performance compared to pure BC aerogel (Figure 3l).^[^ ^48^ ^]^ The abundance of ethyl groups in the hybrid results in a low surface potential, allowing it to donate electrons easily. When the same pressure is applied, porous aerogels experience more deformation than their nonporous counterparts are, resulting in higher capacitance and increased electrical output due to the additional friction layer created on the porous surface.^[^ ^48^ ^]^

Even though TOCN aerogels have found its application in sensing, they used to fail to detect a liquid in a way of low detection limit, fast response, and good robustness. These limitations have been successfully addressed by introducing synthetic polymer‐based aerogels.^[^ ^47^, ^53^ ^]^ For example, the development of polyimide aerogels with remarkable properties (Figure 3m) enables their use as a tribonegative layer in liquid trace analysis. Thanks to 98.01% porosity and ≈220 µm thickness, the polyimide aerogel film facilitates fast response and robust detection of different liquids (Figure 3n,o), achieving a low detection limit of 7 µL.^[^ ^47^ ^]^ Additionally, the utilization of directional freezing in the production of nylon 11 aerogels has eliminated the need for conventional electrical poling processes, resulting in improved dependability and applicability while requiring less energy.^[^ ^53^ ^]^

Another example of aerogel usage in PENG is poly(vinylidene fluoride (PVDF) doped with BaTiO₃/MXene/chitosan (PVDF‐B/MXene/CS) composites, which have demonstrated impressive output performance.^[^ ^61^ ^]^ The addition of MXene layers in these aerogels significantly improves signal transmission, while chitosan (CS) particles serves as internal conductors and connectors. As a result, the device, can reach a peak output current of 62 nA indicating a robust piezoelectric response.^[^ ^61^ ^]^ Additionally, these aerogels achieves a maximum power output of 4.25 nW at an external resistance of 100 MΩ and shows stable voltage output over 1800 operational cycles.^[^ ^54^ ^]^

The study of Montero and colleagues have demonstrated the application of PVDF‐trifluoroethylene P(VDF‐TrFE) porous aerogel in TENG.^[^ ^54^ ^]^ They propose fabricating a P(VDF‐TrFE) porous aerogel film through a three‐step process: freezing the P(VDF‐TrFE) layer dissolved in dimethyl sulfoxide (DMSO), removing the solvent, and freeze‐drying the porous film. The application of the porous film as a triboelectric material and the impact of P(VDF‐TrFE) porous aerogel film polarization are examined by pairing it with three different polymers: polyethyleneterephthalate (PET), polyimide (PI), thermoplastic polyurethane (TPU), and Ecoflex. The device is fully constructed using stretchable materials, such as TPU substrates, Ecoflex, and a P(VDF‐TrFE) porous aerogel as the active layer. This combination achieves optimal performance, producing a voltage of 105.6 ± 10.8 V, a charge of 35.8 ± 2.2 nC, a peak power of 62.8 mW m⁻^2^, and an average power of 9.9 mW m⁻^2^ over 100 tapping cycles at 0.75 Hz.^[^ ^54^ ^]^

Considering the potential of aerogels to function as both PENG and TENG, a PDMS valve structure and bundle‐like porous multi‐walled carbon nanotubes (MWCNTs)/PVDF‐TrFE aerogel bulk are introduced as an innovative, integrated piezo‐tribo hybrid acoustic‐driven nanogenerator (PENG‐TENG).^[^ ^30^ ^]^ The interconnected tiny voids within the porous aerogel (Figure 3p) allow sound waves to travel unevenly between the pore walls and centers, causing the walls to vibrate and rub against each other. This process efficiently converts sound energy into mechanical energy, which is then transformed into electricity (Figure 3q).^[^ ^30^ ^]^ The flexible PDMS valves can also provide a specific supercharging effect and advantageous frequency response properties, simulating the behavior of human eardrum.^[^ ^31^, ^32^ ^]^ The two structures work together to form a developed composite sound resonant cavity, realizing the synergy and enhancement of piezoelectric and triboelectric effects without the need for external structural design. The new structure also provides the integrated device with excellent sound energy harvesting capability while ensuring durability and flexibility.^[^ ^30^ ^]^

While these aerogels demonstrate remarkable performance, challenges persist in their fabrication processes. Issues such as heat exchange, thermal expansion and contraction, and deformation during freeze‐drying need careful consideration. However, innovative methods, such as heterointerface engineering and environmentally friendly freeze‐drying, are beginning to provide solutions to these challenges.

### 3.2 Cryogel

Cryogels are a distinct class of hydrogels, characterized by macropores enclosed within an elastic interconnected network formed through polymerization at subzero temperatures.^[^ ^62^ ^]^ Although cryogels were investigated first in the 1940s, they only gained widespread recognition in 1980 when they were applied for whole‐cell entrapment.^[^ ^63^ ^]^ Thanks to advancements in polymer chemistry, interest in this material has substantially increased in recent years.^[^ ^64^ ^]^

Cryogels offer multiple advantages, including lower cost, simpler fabrication, and faster processing compared to aerogels; for instance, the UV‐irradiation method enables production in just 20 min.^[^ ^63^ ^]^ With their straightforward and rapid fabrication process and high porosity, cryogels are gaining considerable interest for energy production applications.^[^ ^63^ ^]^ Haleem et al. demonstrate this potential by using a cost‐effective UV‐radiation method combined with an in‐situ reduction approach to fabricate a bimetallic hybrid cryogel.^[^ ^55^ ^]^ They identify C03t4 as the optimal monomer concentration, showing nearly double the effectiveness of C06t4, achieving an open circuit voltage (VOC) of 162.26 V and a current density (JSC) of 16.25 mA m⁻^2^, compared to C06t4's VOC of 89.46 V and JSC of 7.33 mA m⁻^2^. Silver and gold nanoparticles are then added to the hybrid bimetallic cryogel film, creating a TENG device in which the cryogel serves as the tribopositive material and PDMS as the tribonegative material. Despite its compact size of 1 × 2 cm^2^, this device delivers an impressive output voltage of 262.14 V, a current density of 27.52 mA m⁻^2^, and a peak power density of 7.44 W m⁻^2^.^[^ ^55^, ^63^ ^]^ These results notably surpass those of devices with individually added gold or silver nanoparticles, achieving VOCs of 213.51 and 233.85 V and JSCs of 23.62 and 24.33 mA m⁻^2^, respectively. The high output is attributed to the film porosity, material roughness, and the enhanced properties provided by the nanoparticles.

**Figure 4:**
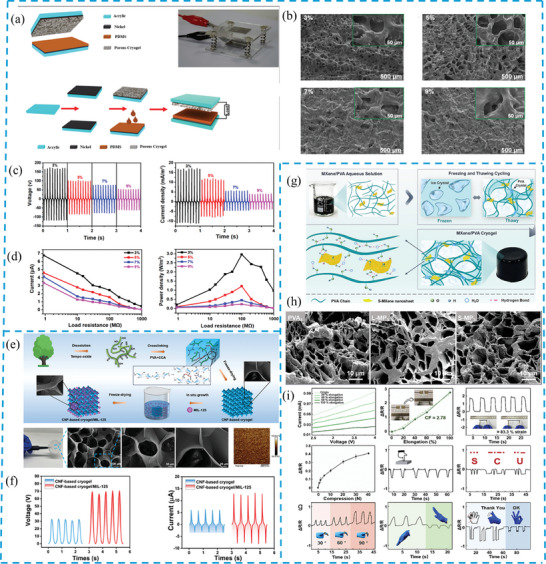
a) Schematic and fabrication process of TENG with porous cryogel and PDMS. b) SEM images of surfaces with various crosslinker concentrations in cryogel films. c) Output voltage and current density with different crosslinker combinations. d) Electric current and power density under varying external loads. e) Schematic of CNF‐based cryogel/MIL‐125 fabrication, with photograph, SEM, and surface potential distribution. f) Output voltage and current of the tribolayer pair. g) MXene/PVA composite cryogels preparation via cyclic freeze‐thawing. h) SEM of PVA3, L‐MP3, and S‐MP3 cryogels. i) Sensor current change under tensile strains, with resistance response to various mechanical signals. (a–d) Reproduced with permission from,^[^ ^56^ ^]^ Copyright 2020, Elsevier Ltd. (e,f) Reproduced with permission from,^[^ ^57^ ^]^ Copyright 2024, Wiley. (g–i) Reproduced with permission from,^[^ ^65^ ^]^ Copyright 2023, Elsevier.

Another team has researched a new method of synthesizing highly porous cryogel films using facile freezing, ultraviolet radiation, and thawing processes.^[^ ^56^ ^]^ Again, cryogel is employed as the tribopositive material, while spin‐coated PDMS serves as the tribonegative material. For the same area of 1 × 2 cm^2^ and the 3% concentration of crosslinker in the film (**Figure** **4**a,b), the voltage output reaches 170 V, and the current density reaches 17.1 mA m^−2^ under short circuit conditions, with an instantaneous power density of 2.95 W m^−2^ (Figure 4c,d).^[^ ^56^ ^]^ This high performance is attributed to the high porosity of the cryogel and the formation of high‐density mechanoradicals associated with the porous structure. Moreover, they have discovered that the crosslinker in the cryogel negatively affects the electrical performance, thus reducing its presence is beneficial. Although these results are lower than those of the previous study, this novel method avoids using silver and gold nanoparticles as additives, making the technique simpler and more cost‐effective.

Alongside the aim of achieving high electrical performance, various applications of cryogel are also under investigation. One example is a respiration‐driven air filter (RAF) developed by Ding et al. (Figure 4e),^[^ ^57^ ^]^ where cryogels made of cellulose‐based electrospun membrane and its composite with MIL‐125 function as tribolayers. Respiration provides the necessary contact‐separation movement for triboelectric phenomenon to occur, generating output as shown in Figure 4f. By continuously generating triboelectric charges to replenish static charges and sustaining an electric field between two layers through breathing‐induced motion, this approach enhances filtration performance.^[^ ^57^ ^]^ Consequently, efficiency is increased to 93.8% for 0.3‐µm particulate matter, and sensing/catalytic degradation is enhanced, with degradation exceeding 20%.^[^ ^57^ ^]^ Moreover, RAF can function as a breathing regulator when used to monitor respiration dynamics.

Despite this progress, problems such as the lack of mechanical strength and difficulty maintaining thermal stability in different conditions still exist. Although some researchers have developed strong and flexible MXene/polyvinyl alcohol (PVA) cryogels (Figure 4g–i),^[^ ^65^ ^]^ which exhibits good mechanical properties and have been used to detect human motion and in smart coatings, several other methods also need to be developed to optimize the fabrication process. Hence, further exploration of cryogels is necessary to adopt them in common electro‐devices widely.

### 3.3 Nano‐Porous Films

Nano‐porous films are thin films with a nanoscale porous structure that can significantly enhance the piezoelectric response of materials due to their high surface area and the ability to amplify mechanical stresses at the nanoscale.^[^ ^66^ ^]^ The introduction of porosity leads to localized mechanical strain around the pores, which distorts the crystal lattice more effectively facilitating the alignment of dipoles within the material.^[^ ^67^ ^]^ As the dipoles align more uniformly under stress, the material exhibits enhanced polarization and, consequently, an improved piezoelectric effect.^[^ ^43^ ^]^ Additionally, the porous structure reduces the material's relative permittivity (ε*_33_*), while maintaining a relatively high piezoelectric charge coefficient (*d_33_*), increasing the piezoelectric voltage coefficient (g*_33_*). This makes the material more suitable for sensing applications due to the higher electric field generated per unit stress.^[^ ^66^ ^]^

**Figure 5:**
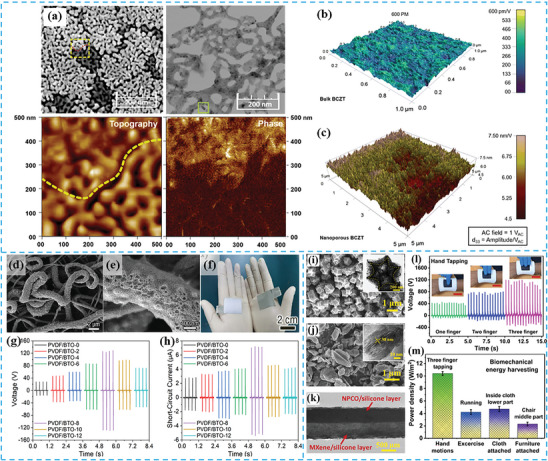
a) Electron micrograph images of nano‐porous BCZT. Amplitude distribution of *d_33_* mapping for b) bulk and c) nano‐porous BCZT. d,e) SEM images of the PVDF/BTO‐8; f) T‐PENG sample; g) Output voltages and h) short‐circuit currents of the T‐PENG based on PVDF/BTO composite films. i) FESEM image of the NPCO powder. j) FESEM image of the MXene powder. The insets show a TEM image of a single particle. k) Schematic illustration of the NDL‐TENG. l) Output voltage of the NDL‐TENG. m) Comparison of the power densities for NDL‐TENG. (a–c) Reproduced with permission from,^[^ ^58^ ^]^ Copyright 2024, The Royal Society of Chemistry. (d–h) Reproduced with permission from,^[^ ^33^ ^]^ Copyright 2022, American Chemical Society. (i–m) Reproduced with permission from,^[^ ^59^ ^]^ Copyright 2022, Elsevier.

Recent studies have utilized a soft‐templating approach to engineer nanoscale strains in nano‐porous polycrystalline ferroelectrics using a nano‐engineered barium calcium zirconium titanate composition, (Ba_0.85_Ca_0.15_) (Ti_0.9_Zr_0.1_) O_3_ (BCZT).^[^ ^58^ ^]^ This method involves the synthesis of nano‐porous BCZT films where the controlled pore wall thickness contributes to highly strained lattice structures while retaining optimal crystal size (<30 nm), essential for achieving high piezoelectricity (**Figure** **5**a). Strain field analysis, obtained through geometric phase analysis and aberration‐corrected high‐resolution scanning transmission electron microscopy (STEM), reveals a strain exceeding 30%.^[^ ^58^ ^]^ The controlled pore wall thickness leads to highly‐strained lattice structures with optimal crystal size, resulting in a giant piezoresponse (*d_33_*) of ≈7500 pm V^−1^, an order of magnitude greater than lead zirconate titanate (PZT) (Figure 5b,c).^[^ ^58^ ^]^ This enhanced piezoelectricity is due to the introduction of the porous structure, which causes strong anisotropic stress on the oxygen octahedron within the BCZT crystal, significantly distorting the crystal lattice and resulting in spontaneous polarization.

Another innovative method involves the nano‐catalyst‐assisted ink spray coating (N‐ISC) method to synthesize boron nitride nanotubes (BNNTs) with a 4.69‐eV optical bandgap.^[^ ^68^ ^]^ This technique facilitates the creation of a flexible, nano‐porous, freestanding BNNT thin film with high flexibility and 91.45% porosity via an easy mechanical peeling process. The piezoelectric *d_33_* coefficient of 90‐nm‐radius BNNTs is significantly enhanced to 41.12 pm V^−1^, attributed to the inner bamboo structure of the nanotubes.^[^ ^68^ ^]^ This nano‐porous bamboo‐type BNNT film also exhibits excellent dielectric properties, with a relative dielectric constant of 5.15, and up to 9.78 for solid types, outperforming cylindrical BNNTs.^[^ ^68^ ^]^ This enhancement makes bamboo‐type BNNT films ideal for high‐precision piezoelectric sensors and intelligent bio‐electronic devices, given their excellent dielectric properties and high porosity.

Despite being mainly based on inorganic ceramics, these films can be combined with polymers to introduce triboelectric properties. Thus, Zhang et al. employed the electrospinning method to obtain a nano‐porous composite thin film combining BaTiO_3_(BTO) (Figure 5d,e), known for its high piezoelectric effect, with PVDF, which exhibits a substantial triboelectric effect.^[^ ^33^, ^34^ ^]^

This composite film has been used as the piezoelectric‐tribonegative layer and the natural rubber film as the tribopositive layer in a TENG‐PENG (Figure 5f). The nano‐porous PVDF/BTO composite film boosts the surface charge density and its overall variation by leveraging the pore dipoles and resulting in improved electrical outputs. The PVDF/BTO‐8‐based TENG‐PENG exhibits a transfer charge density of 38.28 µC m⁻^2^, which is 2.12 times higher than the combined charge density of the PVDF/BTO‐8‐based PENG and the pristine nano‐porous TENG. These 8 wt.% BTO contents give the peak output signals of 124 V and 7.2 µA (Figure 5g,h). Zhang et al.’s nano‐porous PVDF/BTO composite film, created through electrospinning, has demonstrated significant enhancement in electrical output for TENG‐PENG applications.^[^ ^33^, ^34^ ^]^

A similar approach focusing on material synergy is seen in a novel, multifunctional TENG using a double‐layered design with nano‐porous cobalt oxide (NPCO)/silicone and MXene/silicone composites (Figure 5i–k).^[^ ^59^ ^]^ Here, nano‐porous structure of NPCO promotes significant charge accumulation, enhancing the composite's electronegativity fourfold. Meanwhile, an MXene layer introduces charge trapping and transport functions, further boosting electronegativity nine times over traditional materials. Additional hierarchical micro/nanostructures improves hydrophobicity and resilience, while a conductive knitted fabric electrode enables high stretchability (up to 230%) and durability.^[^ ^59^ ^]^ The resulting device achieves a power density of 10.4 W m^−^^2^ — 23 times greater than conventional silicone‐based TENG (Figure 5l,m) — and a sensitivity of 5.82 V kPa^−1^, supporting versatile applications in biomechanical energy harvesting, such as wearable pressure sensors and self‐powered devices. This work underscores the role of nano‐porous structures in advancing the performance and application range of flexible TENG systems.

### 3.4 Micro‐Porous Membranes

Micro‐porous membranes serve as versatile platforms for integrating both PENG and TENG technologies, enabling efficient energy harvesting and sensing capabilities. They are mainly based on organic polymers but can be combined with different inorganic substances to enhance their performance and functionality.^[^ ^69^ ^]^ Key representatives include expanded polytetrafluoroethylene (ePTFE) and PDMS, which are utilized predominantly in TENG due to their flexibility and compatibility with various triboelectric materials. These membranes play a crucial role in applications ranging from wearable sensors to environmental monitoring systems, leveraging their porous structures to capture mechanical energy and convert it into useful electrical power.

**Figure 6:**
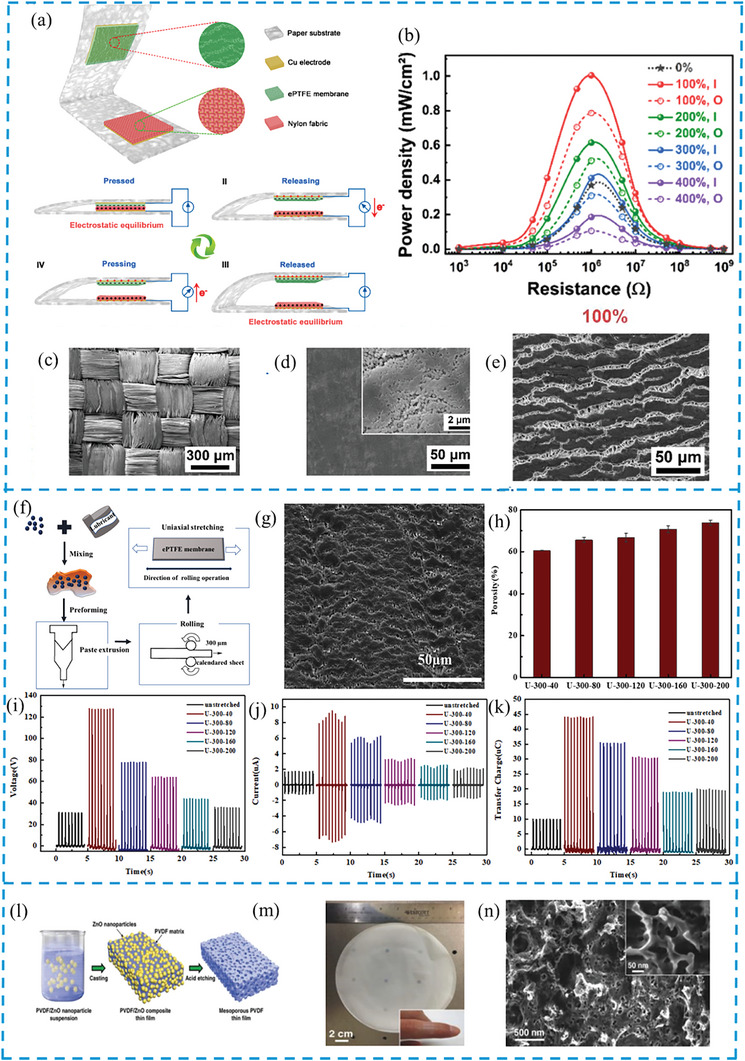
a) Construction of an ePTFE/Nylon‐TENG and its working mechanism. b) Instantaneous power density of ePTFE/Nylon‐TENG on load resistances. c) SEM of nylon fabric. d) SEM of PTFE membrane without expansion. e) SEM of ePTFE membranes with 100% expansion. f) Experimental setup for uniaxially‐stretched ePTFE membranes. g) SEM of ePTFE membrane stretched at 40 °C. h) Porosity of ePTFE membranes stretched at temperatures from 40 to 200 °C. i) Open‐circuit voltage, j) short‐circuit current, and k) transferred charge of ePTFE based TENG membranes stretched to 300% at different temperatures. l) Preparation of mesoporous PVDF thin film. m) Image of porous sponge. n) SEM of obtained porous structure. (a–e) Reproduced with permission from,^[^ ^70^ ^]^ Copyright 2019, Elsevier. (f–k) Reproduced with permission from,^[^ ^69^ ^]^ Copyright 2021, Elsevier. (l–n) Reproduced with permission from,^[^ ^35^ ^]^ Copyright 2014, Wiley.

ePTFE membranes are fabricated by stretching and expanding the PTFE to create microporous structure.^[^ ^70^ ^]^ Microporous structure changes the mechanical properties of the material significantly improving the elasticity and flexibility from the traditional PTFE, which made it suitable for applications such as vascular drafts^[^ ^71^ ^]^ and human motion monitoring.^[^ ^72^ ^]^ Another distinct feature ePTFE membranes offer is the large surface contact area, which increases the charge generation leading to increased charge density on the contact area, which proportionally improves the performance of the TENG.^[^ ^70^, ^73^ ^]^ However, the increase in surface area and triboelectric properties depends on the expansion rate, where excess stretching adversely affects the TENG output.^[^ ^73^ ^]^ According to Zhang's research team, the expansion up to 100% shows an increase in the output electrical signal, 100% reaching the peak performance and gradually dropping further.^[^ ^70^ ^]^ The ePTFE paired with nylon (**Figure** **6**a–e) has stable performances displaying maximum power density of 1.01 mW cm^−2^, with an effective area of 1 cm^2^ and a load resistance of 1 MΩ (Figure 6b). Compared to the non‐porous PTFE/Nylon TENG with maximum power density of 0.37 mW cm^−2^, expanded PTFE shows promising results in the improvement of TENG.^[^ ^70^ ^]^ The same observation is made by the group of Hu et al., who states that the increase in the pore size results in the diminished contact area, thus decreasing the triboelectric output.^[^ ^69^ ^]^ The experiment procedure is given in Figure 6f. The pore size is changed by controlling the ambient temperature at which the PTFE is expanded. A series of stretching under 200 °C and lower temperatures have been conducted to weaken the molecular mobility and get more porosity without compromising the contact area to improve the TENG output. The best TENG output obtained for this group is at uniaxial stretching the PTFE 300% of its original length at 40 °C which shows the open‐circuit voltage, short‐circuit current, and transferred charge reaches ≈120 V, 9.5 µA, and 44 nC, respectively (Figure 6i–k).^[^ ^69^ ^]^ Further improvement has been made by transversely stretching the sample 200% at the same temperature.

The impact of biaxial stretching on the output is negligible; however, it provides greater elasticity, offering enhanced comfort for wearable electronics applications.^[^ ^69^ ^]^ The performance remains stable under various conditions without notable signs of degradation, demonstrating the enhanced durability of these future devices.^[^ ^70^ ^]^ The ePTFE materials can effectively enhance the output performance of ePTFE‐based TENG, thus expanding the application prospects of the material for energy harvesting applications.

PDMS is another suitable material for TENG fabrication in wearable applications due to its flexibility, lightweight nature, non‐flammability, and non‐toxic properties.^[^ ^74^, ^75^ ^]^ Various techniques are applied to introduce a porous structure to PDMS and enhance TENG performance. These methods include curing in a nano‐grass silicon mold to create dispersed porous and micro‐pillar structures, modifying with different foaming agents to achieve multiple pore sizes, mixing with deionized water to form a PDMS slurry for a sponge‐like structure, and curing with 400–600 nm ZnO nanospheres to produce a well‐ordered porous structure in the PDMS film.^[^ ^10^, ^74^, ^76^, ^77^ ^]^ The electrical outputs of the silicon‐molded porous and micro‐pillar structures show a threefold increase in maximum power density for the porous structure, achieving a maximum voltage of 3200 V and a current of 94 µA over an area of 100 cm^2^.^[^ ^77^ ^]^ The optimal foaming agent for PDMS is azodicarbonamide, with peak performance achieved at an 8‐wt.% concentration added to the PDMS. The voltage results are higher by 3.71 times and the current is 3.12 times more than in the flat PDMS‐TENG, with an open‐circuit voltage of 100.38 V and a short‐circuit current of 3.99 µA.^[^ ^74^ ^]^ PDSM with GO flakes made by adding deionized water shows similar output with an output voltage of 140.4 V and a current of 2.57 µA, and proves to be washable without compromising the TENG properties of the material, with high air permeability.^[^ ^10^, ^75^ ^]^ The ordered porous structure achieved by ZnO spheres, which were fully dissolved in hydrochloric acid, shows promising results with a maximum output of 271 V and 7.8 µA at 12% porosity.^[^ ^76^ ^]^ The high performance and flexibility of porous PDMS‐based TENG have great potential in a wide range of applications such as wearable devices, self‐powered sensing, and powering microelectronic devices.^[^ ^75^, ^76^ ^]^

On the other hand, the development of gradient porous lead zirconate titanate (PZT)/PDMS composites has demonstrated remarkable enhancements in piezoelectric properties, with optimized output voltages and currents reaching as high as 152 V and 17.5 µA, respectively.^[^ ^78^ ^]^ The incorporation of transition regions, cellular regions, progressive lamellar regions, and homogeneous lamellar regions in the PZT ceramics, along with the presence of transverse bridges between layers, has facilitated the formation of an interconnected structure, leading to superior piezoelectric coefficients and enhanced electrical outputs.^[^ ^78^ ^]^

### 3.5 Sponge

A sponge is another promising porous structure for both PENG and TENG, offering significant potential due to its highly porous, flexible, and compressible nature. Mao et al. propose a method by casting a mixture of PVDF solution and ZnO nanoparticles onto a flat surface and then etching the mixture with HCl acid solution to remove the ZnO and create the mesoporous sponge–based PVDF thin film as illustrated in Figure 6l–n.^[^ ^35^ ^]^ A flexible, sponge‐like, 3D PENG can be integrated into wearables. The instantaneous output power figures of the suggested generator design are higher than those of other cutting‐edge conventional PVDF‐based nanogenerators.^[^ ^35^ ^]^ The mesoporous sponge‐type PVDF nanogenerators may successfully convert mechanical energy from ambient surface vibrations to electricity by leveraging the weight of the electronic device to increase the amplitude.

Another approach uses electrospinning method to create 3D sponge‐like piezoelectric electrospun nanofiber structures. Thick sponge‐like fiber mats are produced by electrospinning PVDF‐TrFE solutions with added polyethylene oxide (PEO) and lithium chloride.^[^ ^36^ ^]^ The electrospun PVDF‐TrFE/PEO sponge‐like core generators achieve an average peak‐to‐peak voltage of 69.4 V and an instantaneous output power of 40.7 µW cm⁻^2^ under a 1.58 N impact force.^[^ ^36^ ^]^

Along with synthetic polymers, natural sponges, such as wood can be obtained through a simple delignification process.^[^ ^79^ ^]^ The wood sponge‐based nanogenerator can generate a substantial output voltage of 0.69 V, demonstrating stable performance under repeated cyclic compression and environmental sustainability by its biodegradability.^[^ ^79^ ^]^ They can be directly adhered to the surface of an electronic device (such as a cell phone). To increase the output power needed to operate electrical equipment, multiple PVDF nanogenerators can be easily integrated and run synchronically.^[^ ^36^ ^]^

In exploring materials for flexible and high‐output TENG, conductive and elastic sponge‐based TENG (ES‐TENG) have shown great promise for capturing irregular, random mechanical energy.^[^ ^60^ ^]^ Developed by polymerizing aniline on a flexible sponge substrate, this ES‐TENG leverages the flexibility of the sponge and the conductivity of polyaniline nanowires (PANI NWs). The unique combination of a porous structure with PANI NWs enhances the triboelectric layer's contact area, significantly improving output efficiency by flexibly adapting to varying amplitudes and motion directions. The ES‐TENG generates a robust output of 540 V and 6 µA,^[^ ^60^ ^]^ demonstrating potential for integration on diverse flexible surfaces where random motion energy can be harvested. Beyond energy harvesting, this sponge structure has an impressive NH_3_‐sensing capability, making it an effective self‐powered ammonia detector. With sensitivity to detect as low as 1 ppm and a response time under 3 s,^[^ ^60^ ^]^ this ES‐TENG offers dual‐functionality, particularly valuable for environments requiring both power generation and gas sensing. The elasticity, high surface area, and conductivity of the ES‐TENG's sponge structure underscore its value for multifunctional applications, establishing it as a versatile solution for energy and sensor demands in daily life.

## 4 Applications of Porous PENG and TENG

**Figure 7:**
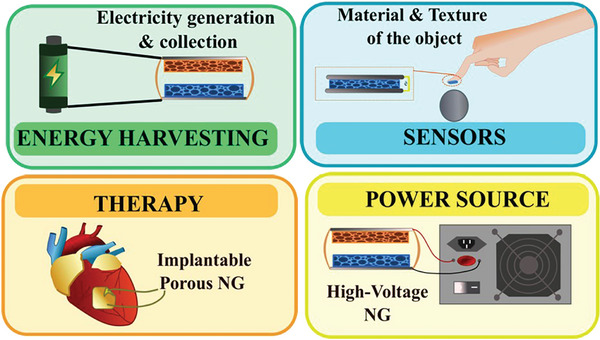
Application of porous PENG and TENG.

Porous PENG and TENG are innovative solutions for harvesting mechanical energy, with applications ranging from wearable technology to environmental energy harvesting and sensing (**Figure** **7**). By incorporating porosity, these nanogenerators achieve enhanced surface area and deformability, leading to improved energy conversion efficiency and higher electrical output.^[^ ^80^ ^]^ This makes them ideal for powering portable electronics and wearable devices, such as health monitoring sensors and fitness trackers, reducing the need for battery replacements and supporting continuous data collection.^[^ ^81^, ^82^, ^83^ ^]^ Their versatility also extends to therapeutic applications and serves as high‐voltage sources.

### 4.1 Energy Harvesting

**Figure 8:**
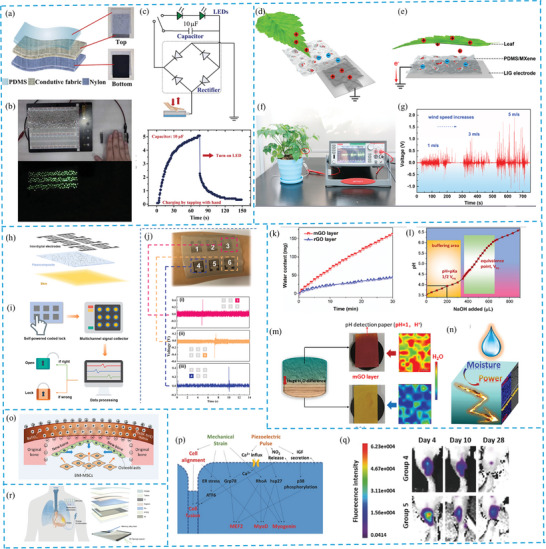
a) Schematic of fabric‐based TENG b) Lighting 180 green LEDs in series c) Commercial LEDs powered by TENG‐charged capacitor d) the device structure for leaf swing energy collection e) left‐attached TENG in single‐electrode f) TENG voltage output with varying wind speeds g) Fabrication and application of 1.5 Ag/S‐PVFE based coded lock. h) Sensor arrays of Ag/S‐PVFE lock i) Operation of flexible coded lock j) Voltage signals by finger input order k) Moisture absorption in H‐mrGO over time l) Titration of mGO with a NaOH standard solution m) H+ Distribution in H‐mrGO Layers n) Moisture‐enabled electricity generation in H‐mrGO o) Electric microenvironment for cartilage regeneration p) Myogenic differentiation and intracellular signaling q) Live Imaging of myogenic cells in muscle regeneration r) Cardiac pacemaker system with implantable TENG. (a–c) Reproduced with permission from,^[^ ^10^ ^]^ Copyright 2022, Elsevier. (d–g) Reproduced with permission from,^[^ ^93^ ^]^ Copyright 2019, Elsevier. (h–j) Reproduced with permission from,^[^ ^53^ ^]^ Copyright 2021, Elsevier. (k–n) Reproduced with permission from [ref], Copyright 2023, Elsevier. (o) Reproduced with permission from,^[^ ^112^ ^]^ Copyright 2016, American Chemical Society. (p,q) Reproduced with permission from,^[^ ^117^ ^]^ Copyright 2017, Wiley (r) Reproduced with permission from,^[^ ^116^ ^]^ Copyright 2019, Nature.

Porous PENG and TENG technologies effectively harvest biomechanical and environmental mechanical energy, powering portable electronics and wearables.^[^ ^84^, ^85^, ^86^ ^]^ They capture energy from daily activities like walking, breathing, and heartbeats, as demonstrated by Yang et al., who have integrated porous PDMS into clothing to light LEDs by storing energy in capacitors (**Figure** **8**a–c).^[^ ^10^ ^]^ Similarly, the TOCN/MoS_2_ aerogel PENG device efficiently charges capacitors, showing the potential of these systems to harness everyday movements as sustainable energy sources.^[^ ^52^ ^]^ Flexible and elastic versions of these devices can be seamlessly incorporated into fabrics or accessories, providing continuous power for wearables without the need for frequent recharging.

In addition to biomechanical energy, porous TENG devices are highly efficient at capturing ambient mechanical energy,^[^ ^87^ ^]^ such as wind and low‐frequency vibrations, outperforming conventional electromagnetic generators in harvesting low‐frequency forces.^[^ ^88^, ^89^, ^90^, ^91^, ^92^ ^]^ For example, porous PDMS/MXene composites adhered to leaves have been used to harness wind energy, (Figure 8d–g),^[^ ^93^ ^]^ while similar systems capture energy during the act of writing, opening the door to applications in tactile sensing and human‐machine interfaces.^[^ ^93^ ^]^ These technologies offer compact, scalable, and cost‐effective alternatives to traditional bulky energy‐harvesting systems, such as wind turbines, making them ideal for various environmental and wearable applications.

### 4.2 Sensing

Self‐powered sensing is a major application of porous PENG and TENG technologies, as they convert mechanical triggers like pressure, strain, and vibrations into electrical signals without needing external power sources or complicated processes.^[^ ^94^, ^95^, ^96^ ^]^ These devices are useful in touch, motion, acceleration, and even chemical sensing applications.^[^ ^97^, ^98^ ^]^ Porous PENG, like translucent porous Ag/S‐PVFE composites made with ZnO nanoparticles (Figure 8h), show enhanced piezoelectric properties due to electrostatic bonding, making them highly sensitive to force and capable of both harvesting energy and detecting mechanical stimuli. This makes them promising for human‐machine interfaces and self‐powered systems, such as coded locks (Figure 8i,j).^[^ ^53^ ^]^ Similarly, PZT ceramic scaffolds with porous structures exhibit high piezoelectric output and sensitivity, offering strong sensing capabilities.^[^ ^99^ ^]^ Acoustic radiation control in GPL‐reinforced FG porous plates, studied using hybrid fuzzy PID strategies, also highlights the potential for sound power management and noise control through porosity adjustments.^[^ ^100^ ^]^ PVA/PVDF composite fiber membranes further extend the application range, exhibiting excellent piezoelectric properties, stress sensitivity, and the ability to monitor body movements and environmental humidity.^[^ ^101^ ^]^ These advancements highlight the potential of PENG for diverse uses, from environmental monitoring to wearable sensors.

Porous TENG, on the other hand, shows a strong correlation between output amplitude and contact pressure, allowing them to infer position, trajectory, and speed of moving objects based on signal profiles. Devices like those made from CNF/PEI aerogel and PVDF nanofibers can detect human motions such as arm bending or foot stepping, with output voltages increasing as bending angles rise.^[^ ^102^ ^]^ Porous PDMS sensors have also been used as pedometers, detecting both heavy footsteps and light finger taps with impressive voltage outputs.^[^ ^10^ ^]^ Beyond human motion, porous TENG are effective in sensing environmental phenomena, such as water droplets, and can even serve as compound detectors by altering output signals in response to specific substances.^[^ ^102^, ^103^ ^]^ For example, H‐mrGO assemblies have shown sensitivity to moisture, producing stable voltages and power outputs (Figure 8k–n).^[^ ^104^ ^]^ These innovations position porous TENG as versatile tools for motion detection, environmental sensing, and more.

### 4.3 Therapy

Porous PENG has gained attention in medical applications due to its ability to influence cellular functions like migration, proliferation, and differentiation through electric fields. These devices generate electric fields triggered by body movements or ultrasounds and convert electrical impulses into mechanical deformations,^[^ ^105^, ^106^ ^]^ enhancing their effects on cellular activities. They have been explored for various therapeutic uses, including wound healing, drug delivery, and neurological stimulation.^[^ ^107^, ^108^ ^]^ Notably, porous graphene‐based pressure sensors show promise in cardiovascular monitoring for health and rehabilitation purposes,^[^ ^109^ ^]^ while piezoelectric materials like pTi and BTi promote bone regeneration by enhancing osteogenesis‐related gene expression and bone formation both in vitro and in vivo.^[^ ^99^ ^]^ Additionally, nanofiber‐aerogel scaffolds stimulated by ultrasound offer promising applications for bone repair (Figure 8o).^[^ ^110^, ^111^, ^112^ ^]^ Ultrasound treatments using PiezoPaint devices have also shown potential in scar reduction, though more research is needed for in vivo applications.^[^ ^111^ ^]^ Piezoelectric biomaterials are also being explored for applications such as cochlear implants, synthetic skin, tissue engineering, and muscle regeneration (Figure 8p,q).^[^ ^113^, ^114^, ^115^ ^]^

TENG is also advancing in medical fields, serving as key power sources for micro/nano self‐powered systems. A significant breakthrough is the implantable TENG, which enables cardiac pacing and sinus arrhythmia correction. This TENG‐based pacemaker forms a symbiotic relationship with the body (Figure 8r), utilizing body‐generated energy to power itself while providing electrical stimulation to regulate heart function.^[^ ^103^, ^116^ ^]^ Despite the potential of these technologies, challenges in clinical translation remain, particularly in terms of biocompatibility and biodegradability.^[^ ^105^ ^]^

### 4.4 Voltage Power Sources

Porous TENG has the potential to be used in high‐voltage (HV) power sources because of its output performance, which displays HV and low current.^[^ ^118^ ^]^ TENG can easily create HV up to 1000 V without complex power converters, making TENG‐based HV power sources more portable and less expensive than traditional HV power sources.^[^ ^119^ ^]^ The high output voltage of TENG has been inventively used to create nano electrospray ionization for mass spectrometric analysis,^[^ ^120^ ^]^ where the limited transferred charges of TENG give control over ion formation that had never been possible. The sliding freestanding TENG generates discrete amounts of charges that are delivered to a micro electrospray ionization emitter for mass spectrometric analysis, ensuring extremely reproducible ionization pulses with the least amount of sample consumption.^[^ ^103^ ^]^ The generated TENG‐triggered electrospray droplet proves that the nano electrospray ionization emitter's onset voltage is achieved.^[^ ^120^ ^]^ The safety of people and equipment is considerably increased by the TENG, which only supplies a low current that is constrained by the charges exchanged in one cycle. Environmental protection and electrostatic actuation have both made use of TENG‐based HV power sources due to their portability, controllability, safety, and high efficiency.^[^ ^12^, ^121^, ^122^ ^]^

## 5 Conclusions and Perspectives

Porous structures have emerged as key elements in advancing PENG and TENG technologies, providing substantial improvements in energy harvesting and sensing by leveraging their high surface area, flexibility, and lightweight properties. These porous materials, such as aerogels, cryogels, nano‐porous films, and sponges, enhance charge accumulation, amplify mechanical stress, and improve electrostatic responses, making them ideal for piezoelectric and triboelectric applications. By addressing efficiency limitations in traditional nanogenerators, porous structures have enabled versatile solutions for energy harvesting in wearable electronics, environmental sustainability, and medical devices.

The integration of porous materials significantly enhances the performance of PENG and TENG. Porous PENG reduce breakdown voltage, increase flexibility, and allow for larger strains, resulting in higher voltage outputs under similar forces. Porous TENG, on the other hand, improve surface roughness and electrostatic induction, leading to robust triboelectric outputs. Aerogels, such as TOCN and MoS_2_ composites, stand out for their ultra‐lightweight nature, high specific surface area, and low thermal conductivity, making them ideal candidates for piezoelectric applications. However, the fragility of aerogels necessitates further innovations to enhance their structural durability. Cryogels, characterized by macro‐porous networks, offer a cost‐effective and rapidly fabricated alternative but face challenges related to mechanical strength and thermal stability. Nano‐porous films and sponges also demonstrate significant energy outputs, with electrospun PVDF‐TrFE and delignified wood sponges showing great potential for wearable electronics and environmental applications.

The scalability and versatility of porous PENG and TENG technologies position them as essential tools for future developments in energy harvesting, particularly in wearable electronics, smart textiles, and IoT devices. Their ability to capture biomechanical energy efficiently from everyday activities and ambient mechanical energy from the environment opens new possibilities for sustainable energy solutions. Moreover, porous nanogenerators show strong potential in medical applications, where they generate electric fields that influence cellular functions, aiding in therapies like cardiovascular monitoring, wound healing, bone regeneration, and drug delivery. Implantable TENG devices, which have already demonstrated success in cardiac pacing, highlight the transformative potential of these technologies in healthcare.

From a materials perspective, ongoing advancements are crucial for realizing the full potential of porous PENG and TENG technologies. Aerogels, especially hybrid composites such as TOCN‐MoS_2_ or PDMS‐coated aerogels, hold promise for future exploration due to their high performance, though further work is needed to improve mechanical strength. Cryogels, particularly MXene/PVA composites, offer an exciting avenue for cost‐effective energy harvesting, but research must continue to address their durability and thermal stability limitations. Nano‐porous films produced through soft‐templating or electrospinning have demonstrated exceptional piezoelectric performance, with PVDF/BTO composites standing out as promising materials for hybrid energy harvesting devices that combine piezoelectric and triboelectric effects. Additionally, natural sponges, like delignified wood, offer sustainable alternatives for flexible and wearable technologies.

The development of hybrid systems that integrate both piezoelectric and triboelectric effects within a single device offers further potential for improving energy conversion efficiency, especially for multi‐functional self‐powered systems. Advances in fabrication techniques, such as heterointerface engineering and environmentally friendly freeze‐drying, will also contribute to optimizing the performance of porous nanogenerators by addressing challenges like heat exchange, thermal expansion, and mechanical deformation during processing.

In summary, porous PENG and TENG technologies represent a promising path forward in energy harvesting, with wide‐ranging applications in consumer electronics, medical treatments, and environmental sustainability. As material innovations and fabrication processes continue to evolve, these technologies are well‐positioned to drive sustainable, self‐powered solutions across a broad spectrum of industries.

## Conflict of Interest

The authors declare no conflict of interest.
